# Diversity of Factors Associated with Physical Inactivity in Patients with Asthma Based on Activity Intensity

**DOI:** 10.3390/jcm15114392

**Published:** 2026-06-05

**Authors:** Keita Murakawa, Tsunahiko Hirano, Keiko Doi, Ayumi Fukatsu-Chikumoto, Yoshikazu Yamaji, Hiroshi Iwamoto, Shintaro Miyamoto, Naoko Higaki, Yoshihiro Amano, Kazuki Anabuki, Mayuka Yamane, Keiji Oishi, Maki Asami-Noyama, Nobutaka Edakuni, Tomoyuki Kakugawa, Kazuto Matsunaga

**Affiliations:** 1 Department of Respiratory Medicine and Infectious Disease, Graduate School of Medicine, Yamaguchi University, Ube 755-8505, Japan; murakawakeita124@gmail.com (K.M.); decem119@yamaguchi-u.ac.jp (K.D.); chiku05@yamaguchi-u.ac.jp (A.F.-C.); yyamaji@yamaguchi-u.ac.jp (Y.Y.); ohishk@yamaguchi-u.ac.jp (K.O.); noyamama@yamaguchi-u.ac.jp (M.A.-N.); edakuni@yamaguchi-u.ac.jp (N.E.); kazmatsu@yamaguchi-u.ac.jp (K.M.); 2 Department of Molecular and Internal Medicine, Graduate School of Biomedical and Health Sciences, Hiroshima University, Hiroshima 734-8551, Japan; hir@hiroshima-u.ac.jp (H.I.); miyamos@hiroshima-u.ac.jp (S.M.);; 3 Department of Internal Medicine, Division of Medical Oncology & Respiratory Medicine, Faculty of Medicine, Shimane University, Izumo 693-8501, Japan; yamano@med.shimane-u.ac.jp; 4 Department of Hematology and Respiratory Medicine, Kochi University, Nangoku 783-8505, Japan; kazukianabuki@kochi-u.ac.jp (K.A.); jm-i-mayuka@kochi-u.ac.jp (M.Y.); 5 Department of Pulmonology and Gerontology, Graduate School of Medicine, Yamaguchi University, Ube 755-8505, Japan; kakugawa@yamaguchi-u.ac.jp

**Keywords:** asthma, activity intensity, desaturation–distance ratio, physical activity, treatable trait

## Abstract

**Background**: The factors contributing to physical inactivity in patients with asthma remain unclear. We aimed to explore the pulmonary and extra-pulmonary factors associated with physical activity (PA) in these patients, with stratification by activity intensity. **Methods**: Patient demographics, Charlson Comorbidity Index, lung function, bronchial and alveolar nitric oxide (NO) levels, six-minute walk test (6 MWT), and PA were cross-sectionally evaluated in healthy participants (*n* = 14) and patients with asthma (*n* = 29). The desaturation–distance ratio (DDR) was measured as an index derived from travel distance and desaturation levels during the 6 MWT. **Results**: Patients with asthma had significantly lower PA than healthy participants, regardless of activity intensity (≥2 metabolic equivalents [METs]: 198 min vs. 240 min, *p* < 0.05; ≥3 METs: 54 min vs. 86 min, *p* < 0.05; ≥4 METs: 10 min vs. 26 min, *p* < 0.01). Extra-pulmonary factors (age, comorbidities, and 6 MW distance) showed higher correlation coefficients with PA as activity intensity increased. Contrastingly, pulmonary factors (asthma severity, airflow limitation, and alveolar exhaled NO) showed lower correlation coefficients with PA as activity intensity increased. The DDR was negatively associated with active time across all activity intensities. **Conclusions**: Our findings suggest that aging and comorbidities are potential limiting factors for moderate-to-vigorous physical activity, whereas asthma severity and airway dysfunction restrict daily life in patients with asthma. Moreover, the DDR could facilitate detection of real-life physical inactivity in patients with asthma.

## 1. Introduction

Despite treatment advances for asthma, it remains a global health problem with a substantial disease burden [1]. The long-term goals of asthma management are accomplishing good symptom control; maintaining normal activity levels; and minimizing future risks such as airflow limitation, exacerbations, and treatment side effects [2]. To achieve these goals and achieve personalized asthma care, the Global Initiative for Asthma (GINA) recommends assessing modifiable factors and adjusting treatable traits in patients with asthma.

Given that physical activity (PA) is a potentially treatable trait in patients with asthma, GINA recommends that patients with asthma engage in regular daily activities for their general health benefits [1]. Patients with asthma have demonstrated decreased PA, which is correlated with poor prognoses, including morbidity, mortality, and medical care utilization [3]. Additionally, increasing PA in patients with asthma has been reported to improve health status and reduce the need for medical care [4,5]. Currently, physical activity assessments in asthma often focus heavily on daily step counts, leaving a gap in our understanding of how various clinical traits limit specific activity intensities ranging from light housework to vigorous exercise. Recent advances in wearable triaxial accelerometry and multidimensional assessment of daily behavior have enabled objective, intensity-stratified measurement of PA in patients with chronic respiratory disease, but evidence in adults with asthma remains limited and rarely distinguishes pulmonary from extra-pulmonary determinants across the full spectrum of activity intensities [6,7,8]. Accordingly, there remains no detailed objective assessment of physical inactivity based on all activity intensities, including daily activities such as housework and indoor walking [9,10,11].

PA in patients with asthma is associated with extra-pulmonary factors, including obesity, advanced age, comorbidities, exercise tolerance, and psychosocial factors, as well as pulmonary factors such as respiratory symptoms and airflow limitation [12]. We hypothesized that pulmonary and extra-pulmonary factors might have different contributions to PA based on activity intensity. Therefore, we aimed to explore the relationship of pulmonary and extra-pulmonary factors with PA in patients with asthma, stratified by activity intensity.

## 2. Methods

### 2.1. Study Design and Participants

This single-center, cross-sectional observational study was approved by the Ethics Committee of Yamaguchi University (Institutional Review Board No. H27-204; approved on 23 March 2016) and registered with the University Hospital Medical Information Network (UMIN-000024749; registered on 8 November 2016). This study used data from outpatients with asthma and healthy participants aged ≥40 years at Yamaguchi University Hospital. Written informed consent was obtained from all participants.

Included patients had an asthma diagnosis confirmed by pulmonary specialists based on the GINA criteria [1]; additionally, they were stable for ≥4 weeks before the study. We excluded participants with poor adherence to asthma therapy or factors that can affect walking and PA, including anemia, trauma, lower limb paralysis, or inadequate inclusion (e.g., complications of malignancy). We collected data regarding patient demographics (sex, age, body mass index, and smoking history), Charlson Comorbidity Index [13], and modified Medical Research Council Dyspnea Scale score. Asthma severity was determined based on the Japanese guidelines for asthma prevention and management [14]. According to asthma severity, patients were divided into the intermittent (mild-intermittent) and persistent (mild-persistent, moderate, or severe) groups. The Charlson Comorbidity Index comprises 19 comorbidities, including heart disease, cerebrovascular disease, respiratory disease, gastrointestinal disease, dementia, paralysis, and lymphoma. Each comorbidity category is weighted on a 1–6 scale, with the total score being used to determine its prognostic impact; specifically, the score is negatively associated with life expectancy. The Charlson Comorbidity Index score was classified into four levels: “Low: 0 points”, “Medium: 1–2 points,” “High: 3–4 points,” and “Very high: ≥5 points” [13]. In the present study, none of the participants scored above four points; accordingly, the score was categorized as follows: “Low: 0 points,” “Medium: 1–2 points,” and “High: 3 points”.

### 2.2. Assessment of Physiological Properties

Lung function was assessed using the CHESTAC-8800 DN type (Chest Ltd., Tokyo, Japan) based on the recommendations of the American Thoracic Society/European Respiratory Society [15]. Exhaled nitric oxide (eNO) levels, including fractional exhaled nitric oxide (FeNO) and alveolar nitric oxide concentration (Calv), were measured using a chemiluminescence-based eNO analyzer (NA-623N; Chest Co. and Kimoto Electric Co., Tokyo, Japan) [16,17,18]. Participants performed a 6 min walk test (6 MWT) to measure the 6 min walk distance (6 MWD) following the American Thoracic Society guidelines [19]. To evaluate the exertional desaturation, peripheral oxygen saturation (SpO_2_) and pulse rate were recorded using a WristOx 2 TM Model 3150 Wrist-worn Pulse Oximeter (Nonin Medical Inc., Plymouth, MN, USA). These data were analyzed using WristOx2 software (Star Product Co., Ltd., Tokyo, Japan); the exact software version could not be identified from the archived study records.

The desaturation area (DA) for the 6 MWT was obtained as the sum of the differences between 100% and the recorded SpO_2_ sampled every second [20]. The desaturation–distance ratio (DDR) was defined as the DA to 6 MWD ratio (DDR as [100 − SpO_2_] × second (DA)/6 MWD). All participants wore an Active Style Pro HJA-750C^®^ (Omron Healthcare Co., Ltd., Kyoto, Japan) with triaxial acceleration, with continuous PA assessment for 2 weeks. Metabolic equivalents (METs) were estimated using the built-in algorithm of the Active Style Pro HJA-750C® triaxial accelerometer (Omron Healthcare Co., Ltd., Kyoto, Japan), which is based on activity-type-specific regression equations derived from triaxial acceleration and METs measured by indirect calorimetry using the Douglas bag method. The HJA has been validated for assessing PA in patients with chronic obstructive pulmonary disease (COPD) [21]. Data regarding PA were extracted from the last three full days (out of the 2 weeks), with exclusion of rainy days, weekdays, holidays, and the first and last days, as previously described [22]. This sampling strategy was selected to minimize confounding variations caused by weather (rainy days), non-routine schedules (holidays) and occupational activity (weekdays), and to allow time for habituation to the accelerometer after the initial wear period; the procedure has been used previously in patients with chronic respiratory disease [22]. In the present study, a total PA duration of ≥2 METs was estimated as the time for activities of daily life; furthermore, PA duration of ≥3 METs and ≥4 METs were estimated as those of active life [23].

### 2.3. Statistical Analysis

Continuous and categorical variables are presented as medians (interquartile range [IQR] and numbers, respectively. Continuous variables were compared using the Kruskal–Wallis and Wilcoxon’s rank sum tests, while categorical data were compared using Pearson’s chi-squared test. Spearman’s rank correlation test was used to analyze the relationship between PA and clinical parameters. Multivariate logistic regression was not performed because the sample size was too small to provide statistical power for multivariate analysis. Given the modest sample size, the present correlation analyses are considered exploratory and hypothesis-generating, with limited power to detect small effect sizes (|ρ| < 0.30) or to support multivariable adjustment; the reported associations should therefore be interpreted as preliminary and confirmed in larger cohorts. All statistical analyses were performed using JMP Pro v16.0.0 (SAS Institute Inc., Cary, NC, USA). Statistical significance was set at *p* < 0.05.

## 3. Results

Table 1 shows the baseline patient characteristics. We included 43 participants (14 healthy participants and 29 patients with asthma). Compared with healthy participants, patients with asthma were more likely to be male and obese; however, there were no significant between-group differences in age and smoking history. Patients with asthma were characterized by more severe airflow limitation (%FEV1, 89.1% vs. 107.4%; *p* < 0.005) and higher levels of airway inflammation (FeNO, 29.3 ppb vs. 18.8 ppb; *p* < 0.05, Calv, 7.4 ppb vs. 4.7 ppb; *p* < 0.005) than healthy participants. Additionally, patients with asthma had more comorbidities than healthy participants. There was no significant between-group difference in the median 6 MWD; however, patients with asthma had a significantly greater DDR than healthy participants (4.65 ([100 − SpO_2_] × second/6 MWD) vs. 3.28 ([100 − SpO_2_] × second/6 MWD) (*p* < 0.01).

**Figure 1:**
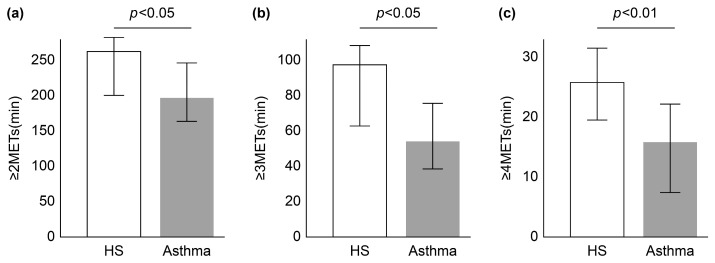
Physical activity in patients with asthma (*n* = 29) and healthy participants (*n* = 14). (**a**) Comparison of physical activity duration at ≥2 METs (min/day). (**b**) Comparison of physical activity duration at ≥3 METs (min/day). (**c**) Comparison of physical activity duration at ≥4 METs (min/day). HS, healthy participants; METs, metabolic equivalents; min/day, minutes per day. Data are presented as median with 25th–75th percentiles. Between-group differences were analyzed using the Wilcoxon rank sum test.

As shown in Figure 1, PA at all activity intensities was lower in patients with asthma than in healthy participants (≥2 METs: 198 min vs. 240 min, *p* < 0.05; ≥3 METs: 54 min vs. 86 min, *p* < 0.05; ≥4 METs: 10 min vs. 26 min, *p* < 0.01).

Next, we analyzed the correlation between PA stratified by activity intensity and the clinical parameters (Table 2). Extra-pulmonary factors (age, Charlson Comorbidity Index, and 6 MWD) showed a higher correlation coefficient with PA as the activity intensity increased.

**Figure 2:**
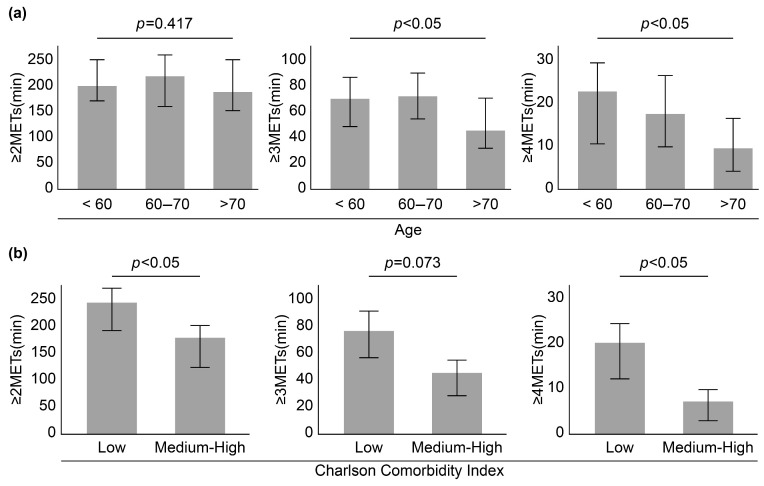
Relationship between extra-pulmonary factors and physical activity at each activity intensity in patients with asthma (*n* = 29). (**a**) Comparison of physical activity duration (min/day) at ≥2, ≥3, and ≥4 METs across age strata (<60, 60–70, >70 years). (**b**) Comparison of physical activity duration (min/day) at ≥2, ≥3, and ≥4 METs by Charlson Comorbidity Index category. The Charlson Comorbidity Index was categorized as low (score: 0), medium (score: 1–2), and high (score: 3); the medium and high categories were combined as “Medium-High” in panel (**b**) owing to the small number of patients with high scores. METs, metabolic equivalents; min/day, minutes per day. Data are presented as median with whiskers extending to the 25th–75th percentiles. Differences across age strata were analyzed using the Kruskal–Wallis test, and differences between the two Charlson Comorbidity Index groups were analyzed using the Wilcoxon rank sum test.

Figure 2 shows the relationship between extra-pulmonary factors and physical activity at each activity intensity. PA at higher activity intensity decreased with older age and higher comorbidities (age; *p* < 0.01 in ≥4 METs, Charlson Comorbidity Index; *p* < 0.05 in; ≥4 METs). Opposite trends were observed for pulmonary factors (asthma severity and lung function). Additionally, the peripheral pulmonary factors (%V50, %V25, and Calv) showed similar trends as the aforementioned pulmonary factors.

**Figure 3:**
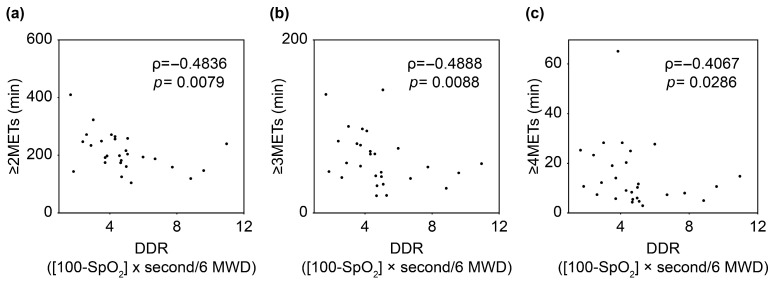
Relationship between the desaturation–distance ratio (DDR) and physical activity at each activity intensity in patients with asthma (*n* = 29). (**a**) Correlation between physical activity duration at ≥2 METs (min/day) and DDR. (**b**) Correlation between physical activity duration at ≥3 METs (min/day) and DDR. (**c**) Correlation between physical activity duration at ≥4 METs (min/day) and DDR. METs, metabolic equivalents; min/day, minutes per day; 6 MWD, six-minute walk distance; DDR, desaturation–distance ratio ([100 − SpO_2_] × second/6 MWD); ρ, Spearman’s rank correlation coefficient. Each data point represents an individual patient. Associations were analyzed using Spearman’s rank correlation test.

Contrastingly, the DDR was significantly negatively correlated with PA at all activity intensities (Table 2 and Figure 3).

## 4. Discussion

### 4.1. Principal Findings

We observed that PA, regardless of activity intensity, was limited in patients with asthma compared with healthy participants. Moreover, PA at higher activity intensity was significantly correlated with extra-pulmonary factors, including aging and comorbidity. Contrastingly, PA at lower intensity was associated with pulmonary factors, including asthma severity, airflow limitation, and peripheral airway impairment. These findings suggest that extra-pulmonary morbidities are associated with limitations of active behavior in patients with asthma and that pulmonary dysfunction can be a potential limiting factor for activities of daily living. Furthermore, we found that DDR was significantly correlated with PA at all activity intensities. This suggests that DDR could be a novel biomarker for detecting physical inactivity in patients with asthma.

### 4.2. Comparison with Other Studies

In patients with asthma, PA is a factor of interest since modifiable traits must be detected in precision medicine [24,25]. However, the relationship of pulmonary and extra-pulmonary factors in asthma with PA classification based on activity intensity remains unclear. In our study, PA at high and low activity intensity was significantly correlated with extra-pulmonary and pulmonary factors, respectively, which is consistent with previous reports [25]. This suggests that the pathophysiology of moderate-to-vigorous activity in patients with asthma is related to extra-pulmonary factors, with physical training and counseling being potential therapeutic targets [26]. Although the median 6 min walk distance (6 MWD) did not differ significantly between patients with asthma and healthy participants (450 m vs. 474 m, *p* = 0.092), the calculated mean walking velocity tended to be lower in the asthma group (75.0 m/min vs. 79.0 m/min), and the asthma group showed a consistently shorter walking distance, a significantly higher desaturation–distance ratio (DDR, 4.65 vs. 3.28 [100 − SpO_2_] × second/6 MWD, *p* < 0.01), and a significantly lower accelerometer-derived PA at every activity intensity. Considered together, these findings indicate that asthma impairs not only the absolute walking distance achievable during a standardized exercise test, but, more importantly, the efficiency of pulmonary gas exchange during exercise (greater desaturation per meter walked) and the translation of exercise tolerance into real-life PA. We also observed a clear age gradient: patients aged >70 years showed significantly lower ≥3 METs and ≥4 METs activity than patients aged <60 years and 60–70 years (Figure 2a, *p* < 0.05), suggesting that age-related comorbidities (cardiovascular disease, diabetes mellitus, cerebrovascular disease and chronic kidney disease) progressively limit moderate-to-vigorous PA. From a practical standpoint, these findings can inform a stratified intervention strategy: pulmonary rehabilitation, walking programs, weight management and control of cardiometabolic comorbidities should be prioritized when limitations predominantly affect moderate-to-vigorous activity (≥3–4 METs), whereas intensification of asthma control (anti-inflammatory therapy and small-airway-targeted treatment) is likely to have the greatest impact when limitations affect activities of daily living (≥2 METs). Psychosocial factors such as anxiety, depression and low motivation are also likely to attenuate moderate-to-vigorous PA and may explain part of the residual variance in our extra-pulmonary correlations [27,28].

Reducing time spent in sedentary behaviors has been shown to improve health status and life expectancy [29]. Further, improvements in airflow limitation can ameliorate PA levels in patients with COPD [30]. This suggests that controlling pulmonary factors may ameliorate limitations in the activities of daily living among patients with asthma. Moreover, lower-intensity PA was more significantly associated with peripheral airway impairments, including %V50, %V25, and Calv, than with central airway impairment. This indicates that small airway dysfunction may strongly contribute to the pathophysiology of daily life behaviors in patients with asthma. At low intensity (≥2 METs), which corresponds to most activities of daily living such as housework, indoor walking and self-care, reductions in PA were most strongly correlated with pulmonary factors—in particular asthma severity, airflow limitation, peripheral airway impairment (%V50, %V25 and %V25/Ht) and alveolar exhaled nitric oxide. This pattern implies that optimization of asthma control, with a specific emphasis on peripheral airway inflammation, may have the greatest immediate impact on the everyday behavior of patients with asthma.

**Figure 4:**
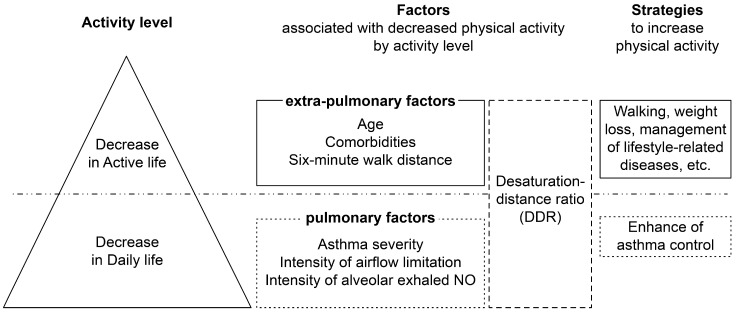
Strategies to increase physical activity according to physical activity levels in patients with asthma. Activities in daily life are divided into daily activity level and activity level. Furthermore, extra-pulmonary factors (age, comorbidities, and walking distance in 6 min) and pulmonary factors (asthma severity, intensity of airflow limitation, and intensity of alveolar exhaled nitric oxide [NO]) are involved in the reduction at each level. Extra-pulmonary factors can be addressed by walking, weight loss, and lifestyle disease management, whereas pulmonary factors can be addressed by enhancing asthma control. DDR [desaturation–distance ratio] is involved in various activities and can be used to screen for decreased activity levels.

It remains unclear whether DDR, which reflects dynamic gas exchange impairment, is related to objectively measured PA in patients with chronic respiratory diseases such as asthma, COPD, and interstitial lung disease. The DDR is computed as the cumulative desaturation area (the integral of [100 − SpO_2_] sampled every second during the 6 MWT) divided by the 6 MWD, and therefore quantifies how much desaturation a patient accumulates per meter walked. Compared with the 6 MWD or the nadir SpO_2_ alone, the DDR integrates both the duration and the depth of desaturation and reflects the overall efficiency of pulmonary gas exchange during exercise, making it a sensitive bedside index of exertional hypoxemia. This study provides novel evidence indicating that desaturation during exertion is important for PA and that DDR could be a promising biomarker of real-life physical inactivity in patients with asthma. Exercise induces bronchospasm (exercise-induced bronchospasm [EIB]) in many patients with asthma, which leads to hypoxemia. Since EIB is more likely to be induced by exercise, the relationship between DDR and PA at higher activity intensities may be attributable to EIB. In our study, the correlation coefficient between DDR and PA increased as activity intensity decreased. Patients with asthma may present air trapping and lung hyperinflation, which depends on the severity of airway narrowing [31]. Moreover, this phenomenon may result from small airway constriction and inflammation. This may explain the association between PA at a lower intensity and DDR. As aforementioned, DDR appears to be associated with PA through different mechanisms at high and low intensities. Specifically, the absolute correlation coefficient between DDR and PA increased as activity intensity decreased (≥2 METs: ρ = −0.484; ≥3 METs: ρ = −0.478; ≥4 METs: ρ = −0.407). This is not paradoxical: at high intensity, EIB and ventilation–perfusion mismatch under increased oxygen demand dominates the DDR–PA association, whereas at low intensity the same patients are limited by chronic small airway dysfunction, air trapping and lung hyperinflation, which restrict even light daily activities. Thus, the relative contribution of resting and sub-maximal pulmonary impairment becomes more visible as the exercise stimulus weakens. From a clinical standpoint, the DDR can be obtained routinely during the standard 6 MWT and could therefore serve as a simple bedside screening tool to identify physically inactive patients who may benefit from intensified asthma control or earlier evaluation of cardiometabolic comorbidities. Therefore, DDR may allow screening for patients with inactive asthma. Figure 4 shows the hypothetical relationship between DDR and PA as well as a diagram of strategies for increasing PA by activity intensity. Patients with poor DDR may be less active, which may inform a review of the underlying asthma control and a closer examination of comorbidities.

### 4.3. Strength and Limitations

This is the first study to investigate the relationship between objectively measured PA based on activity intensity and asthma-related factors. Given that the underlying mechanisms of physical inactivity in patients with asthma may differ according to activity intensity, it is important to establish specific therapeutic strategies for PA according to activity intensity in asthma management. Briefly, this evidence may inform precision medicine when promoting PA in patients with asthma. Moreover, we have expanded the evidence indicating that the DDR could be a novel biomarker for effectively and broadly identifying physical inactivity in patients with asthma. However, our study has several limitations. First, we did not consider several variables that affect daily activities, including pet ownership and psychological factors. Additionally, anxiety and depression are associated with physical health status and PA [27,28]. Accordingly, future studies should assess the impact of these factors. Second, we could not establish the causal relationship between PA limitations and associated factors. Clarifying this relationship may inform elucidation of whether interventions for pulmonary and extra-pulmonary factors are related to the improved health status of patients with asthma. Finally, since this was a single-center cross-sectional study, uncontrolled bias cannot be excluded. Therefore, further large-scale studies are warranted to validate our findings. Several additional caveats should be highlighted. The cross-sectional design precludes any inference about causality or directionality of the associations reported, and the single-center tertiary-care recruitment may have introduced a selection bias toward more severe and/or more adherent patients, which limits the generalizability of our findings. The modest sample size (*n* = 29 patients with asthma) further restricts statistical power and necessitates caution in interpreting the magnitude of the reported correlations; PA durations are reported as median minutes per day to allow direct interpretation of these magnitudes. Accordingly, future longitudinal cohorts and randomized interventional studies in larger, multicenter populations are warranted to demonstrate whether targeted management of extra-pulmonary comorbidities (e.g., cardiovascular risk factors, obesity, sarcopenia) or intensification of asthma control directly increases moderate-to-vigorous activity capacity in patients with asthma.

## 5. Conclusions

We demonstrated that aging and comorbidities are potential limiting factors for moderate-to-vigorous physical activity, whereas asthma severity and airway dysfunction, including small airways, restrict daily life in patients with asthma. Moreover, the DDR could be a support tool for detecting real-life physical inactivity in patients with asthma.

## Figures and Tables

**Table 1:** Characteristics of the study participants.

*p*-Value	Asthma (*N* = 29)	Healthy Participants (*N* = 14)	
0.0647	17/12	4/10	Sex [male/female]
0.2481	69 (74–58)	62 (70–56)	Age (years)
0.0737	24.9 (26.5–21.9)	22.4 (24.2–20.2)	Body mass index (kg/m^2^)
0.1964	15/12/2	11/2/1	Smoking history [non/ex/current]
0.0071	7 (0–23.9)	12.9 (0–5)	Smoking history [pack-years]
NA	8/7/6/8	–	Asthma severity [mild (intermittent/persistent)/moderate/severe]
NA	19 (76)	–	ICS/LABA, *n* (%)
NA	8 (32)	–	LTRA, *n* (%)
NA	9 (6)	–	LAMA, *n* (%)
NA	3 (12)	–	Any biologics, *n* (%)
0.0376	19/9/1	14/0/0	Charlson Comorbidity Index [low/medium/high]
0.2060	12/13/3/1	8/6/0/0	mMRC [0/1/2/3]
			Pulmonary function tests
0.0037	89.1 (72.6–104.8)	107.4 (97.0–114.8)	%FEV1 (%)
0.1199	100.6 (88.5–115.3)	108.2 (83.2–122.5)	%FVC (%)
0.0049	71.9 (65.1–76.9)	78.2 (74.6–88.4)	FEV1/FVC (%)
0.0008	64.8 (36.3–78.1)	98.6 (73.4–131.1)	%V50 (%)
0.0002	36.2 (21.3–49.6)	72.7 (56.4–109.7)	%V25 (%)
0.0001	25.6 (14.8–31.6)	52.1 (39.2–69.9)	%V25/Ht, (%)
0.0249	29.3 (18.9–62.5)	18.8 (14.5–25.9)	FeNO (ppb)
0.0034	7.4 (4.8–10.1)	4.7 (2.3–5.7)	Calv (ppb)
			Six-min walk test
0.0923	450 (372–491)	474 (464–480)	6 MWD (m)
0.0051	4.65 (3.61–5.17)	3.28 (2.43–3.60)	DDR ([100 − SpO_2_] ×second/6 MWD)

ICS, inhaled corticosteroid; LABA, long-acting beta-2 adrenergic agonist; LTRA, leukotriene receptor antagonist; LAMA, long-acting muscarinic antagonist; mMRC, modified Medical Research Council; %, percent predicted; FEV1, forced expiratory volume in one second; FVC, forced expiratory capacity; V50, flow at 50 of vital capacity; V25, flow at 25 of vital capacity; Ht, height; FeNO, exhaled nitric oxide fraction; Calv, alveolar NO concentration; 6 MWD, six-min walk distance; DDR, desaturation–distance ratio. All values are median (25th–75th percentiles). NA = not available. The Wilcoxon’s rank sum test is employed to measure the differences in each group.

**Table 2:** Correlation between physical activity and clinical variables in patients with asthma.

≥4 METs		≥3 METs		≥2 METs		
*p*-Value	ρ	*p*-Value	ρ	*p*-Value	ρ	
<0.0001	−0.7131	0.0024	−0.5420	0.0817	−0.3287	Age
0.8521	−0.0362	0.6816	−0.0796	0.6816	−0.0796	Body mass index
0.7877	0.0523	0.9238	0.0186	0.0994	−0.3121	Smoking history
0.2331	−0.2285	0.0909	−0.3197	0.0144	−0.4496	Asthma severity
0.0028	−0.5340	0.0241	−0.4178	0.016	−0.4434	Charlson Comorbidity Index
0.5644	0.1116	0.77	0.567	0.6501	−0.0879	mMRC
						Pulmonary function tests
0.1562	0.2703	0.0501	0.3672	0.0185	0.4345	%FEV1
0.2631	0.2148	0.2837	0.206	0.1711	0.2612	%FVC
0.189	0.251	0.081	0.3294	0.0055	0.5018	%V50
0.3732	0.1717	0.1159	0.2983	0.0039	0.5191	%V25
0.0361	0.3907	0.003	0.5315	0.0003	0.6271	%V25/Ht
0.1736	0.2597	0.6601	0.0853	0.7596	−0.0594	FeNO
0.9433	0.0138	0.3826	−0.1684	0.0285	−0.4068	Calv
						Six-min walk test
0.0026	0.5376	0.0074	0.4869	0.1523	0.2728	6 MWD
0.0286	−0.4067	0.0088	−0.4777	0.0079	−0.4836	DDR

Abbreviations: mMRC, modified Medical Research Council; %, percent predicted; FEV1, forced expiratory volume in one second; FVC, forced expiratory capacity; V50, flow at 50 of vital capacity; V25, flow at 25 of vital capacity; Ht, height; FeNO, exhaled nitric oxide fraction; Calv, alveolar NO concentration; 6 MWD, six-min walk distance; DDR, desaturation–distance ratio ([100 − SpO_2_] × second/6 MWD); METs, metabolic equivalents. Data were analyzed by Spearman’s rank correlation coefficient. ρ, Spearman rank correlation.

## Data Availability

All data relevant to this analysis are included in this article; the de-identified individual-participant dataset is available from the corresponding author upon reasonable request, subject to applicable institutional and ethical regulations.

## Abbreviations

The following abbreviations are used in this manuscript:

Calv	Alveolar nitric oxide concentration
DDR	Desaturation–distance ratio
eNO	Exhaled NO
FeNO	Fractional exhaled nitric oxide
IQR	Interquartile range
METs	Metabolic equivalents
PA	Physical activity
